# A Novel Microshear Geometry for Exploring the Influence of Void Swelling on the Mechanical Properties Induced by MeV Heavy Ion Irradiation

**DOI:** 10.3390/ma15124253

**Published:** 2022-06-15

**Authors:** Jonathan G. Gigax, Matthew R. Chancey, Dongyue Xie, Hyosim Kim, Yongqiang Wang, Stuart A. Maloy, Nan Li

**Affiliations:** 1 Operational Readiness and Implementation, Los Alamos National Lab, Los Alamos, NM 87545, USA; 2 Center for Integrated Nanotechnologies, Los Alamos National Lab, Los Alamos, NM 87545, USA; dongyue@lanl.gov (D.X.); yqwang@lanl.gov (Y.W.); nanli@lanl.gov (N.L.); 3 Materials Science at Radiation and Dynamics Extremes, Los Alamos National Lab, Los Alamos, NM 87545, USA; mchancey@lanl.gov (M.R.C.); hkim@lanl.gov (H.K.); stuart.maloy@pnnl.gov (S.A.M.); 4 Reactor Materials and Mechanical Design, Pacific Northwest National Lab, Richland, WA 99354, USA

**Keywords:** microshear, micromechanical testing, nanoindentation, radiation effects, ion irradiation, void swelling

## Abstract

Small disks are often the specimen of choice for exposure in nuclear reactor environments, and this geometry invariably limits the types of mechanical testing that can be performed on the specimen. Recently, shear punch testing has been utilized to evaluate changes arising from neutron irradiation in test reactor environments on these small disk specimens. As part of a broader effort to link accelerated testing using ion irradiation and conventional neutron irradiation techniques, a novel microshear specimen geometry was developed for use with heavy-ion irradiated specimens. The technique was demonstrated in pure Cu irradiated to 11 and 110 peak dpa with 10 MeV Cu ions. At 11 peak dpa, the Cu specimen had a high density of small voids in the irradiated region, while at 110 peak dpa, larger voids with an average void swelling of ~20% were observed. Micropillar and microshear specimens both exhibited hardening at 11 dpa, followed by softening at 110 dpa. The close alignment of the new microshear technique and more conventional micropillar testing, and the fact that both follow intuition, is a good first step towards applying microshear testing to a wider range of irradiated materials.

## 1. Introduction

The need to understand the microstructural changes expected in nuclear reactor environments is critical to charting the lifetime and performance of fuel cladding and structural materials. Commitments to test reactors and subsequent analysis are lengthy at higher damage levels and not always a viable route for alloys undergoing rapid development. Ion irradiation is a popular technique for simulating damage to the microstructure caused by neutron irradiation and has been used extensively as a screening tool for a variety of systems [1,2,3,4,5,6]. It is important to note, however, that a range of neutron atypical effects are present during ion irradiation and should be minimized or avoided [7,8,9,10,11,12]. Some of these effects, notably the short ion range, can present complications when evaluating the mechanical properties of ion irradiated materials.

Nanoindentation has been brought to the forefront of this effort, although other techniques, such as micropillar compression, have been employed when using light ions that achieve deeper penetration depths [13,14,15,16,17,18,19,20]. Evaluating the mechanical property changes arising from heavy ion irradiation, often used in radiation-induced hardening and swelling studies, is especially challenging due to the very shallow ion range. Focused ion beam (FIB) milling of mechanical test structures (i.e., pillars, cantilevers, tensile bars) in these regions is often restricted to sub-micron specimens or requires sample orientations that limit access to the vast majority of the ion-incident surface.

In this study, we showcase a new mechanical testing geometry built upon our earlier studies with microshear specimens [21]. Here, a structure is etched using FIB that, when compressed, places a well-defined region under shear stress for simple shear deformation. This particular structure can be placed in grains and oriented to confine the shear entirely within the prescribed shear region. Further, the dimensions of the shear region can be carefully controlled to contain selective parts of the ion range otherwise difficult to achieve with more conventional structures.

The motivation for this study comes, in part, from the relative lack of shear testing of ion irradiated specimens. The ability to isolate and load interfaces and boundaries offers unique opportunities to study the deformation behavior otherwise inaccessible by conventional techniques. More importantly, studying the shear response of heavy ion irradiated materials parallels ongoing efforts with neutron irradiated materials. Typically, small disks are irradiated in test reactors to study microstructural changes. A recent study by Eftink et al. showed the mechanical behavior of these small, irradiated disks when subjected to shear punch testing [22]. As part of a broader effort to link the mechanical properties of heavy ion irradiated specimens to those after neutron irradiation, we conducted microshear compression testing of heavy ion irradiated Cu specimens.

## 2. Materials and Methods

Single crystal Cu (110) specimens, purchased from MTI Corporation (MTI Corporation, Richmond, CA, USA), were irradiated with 10 MeV Cu^4+^ ions at 400 °C to peak damage levels of 11 and 110 peak dpa (referred to as 11 dpa and 110 dpa), as calculated by SRIM using a displacement energy of 30 eV [23]. Damage levels were computed using $dpa=v\frac{\Phi }{\rho }$, where *v* is the vacancies/cm-ion output from SRIM, *Φ* is the ion fluence (ions cm^−2^), and *ρ* is the ion incident target density (atoms cm^−3^). For this experiment, the ion fluences at 11 and 110 peak dpa were 7.4 × 10^15^ cm^−2^ and 7.4 × 10^16^ cm^−2^, respectively. Although samples were mounted with a tilt of less than 2°, SRIM–based estimates for damage levels are made for polycrystalline/amorphous targets and are reported in this study.

Transmission electron microscopy (TEM), micropillar, and microshear specimens were prepared using a Thermo Fisher Helios (Thermo Fisher Scientific, Hilsboro, OR, USA) dual focused ion beam/scanning electron microscope (SEM). TEM foils were prepared using a conventional lift-out technique, thinned with 30 keV Ga ions, and polished to a final thickness of less than 150 nm using a 5 keV beam. A Thermo Fisher Titan TEM (Thermo Fisher Scientific, Hilsboro, OR, USA) operated at 300 kV was used to characterize the irradiated Cu specimens, and conventional electron energy loss spectroscopy (EELS) was used to measure the thickness of the TEM lamella. A machine learning-driven analytical tool built on the Detectron2 (Mask R-CNN based) software package was used to generate statistics on over 1000 voids per irradiation condition [24].

All micropillar and microshear specimens were compressed on a Bruker PI-85 (Bruker, Eden Prairie, MN, USA) equipped with a 10 µm flat punch tip. Micropillar specimens were fabricated with dimensions of 1 µm × 1 µm × 2µm (width × thickness × height) and compressed at a rate of 4 nm/s. Microshear specimens were fabricated with shear region dimensions of 1 µm × 1 µm × 1.5 µm (width × thickness × height) and compressed at a rate of 30 nm/s. In order to access shallow depths with the microshear specimen, a W cap was deposited prior to milling. Nanoindentation was performed using a Keysight G200 (Keysight Technologies, Santa Rosa, CA, USA) equipped with a Berkovich tip at a constant strain rate of 0.02 1/s to a final displacement of 1000 nm. Continuous stiffness measurements at a frequency of 45 Hz and 2 nm were used to extract hardness and modulus values determined using the Oliver-Pharr method [25]. All Cu specimens were oriented similarly to avoid the influence of orientation on the measured hardness and modulus values.

## 3. Results and Discussion

**Figure 1:**
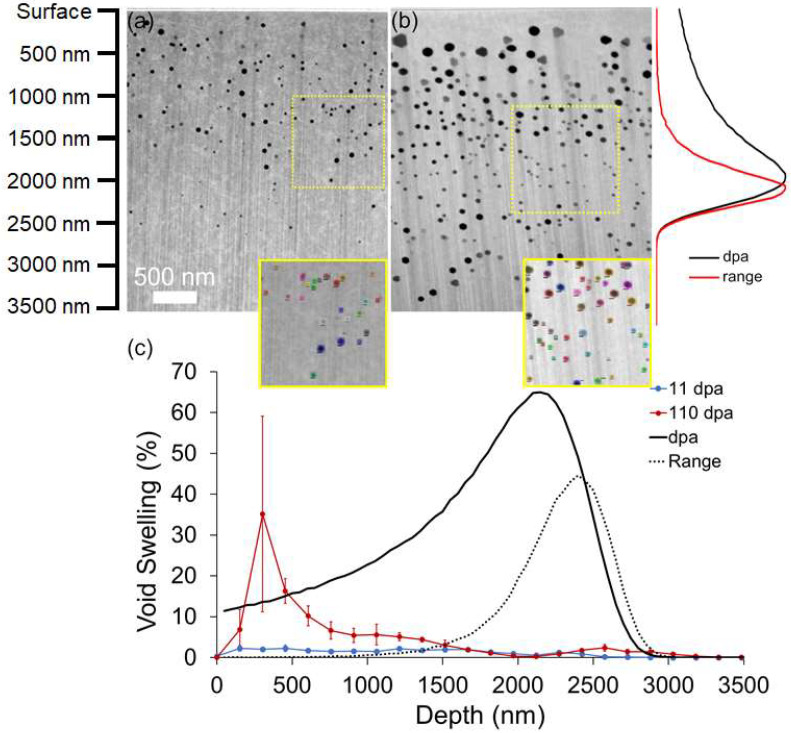
STEM micrographs of Cu(110) irradiated to (**a**) 11 and (**b**) 110 dpa, respectively. The inset in (**a**,**b**) shows the segmentation accuracy of the ML-based analytical tool. (**c**) Void swelling profile as a function of depth below the ion incident surface.

Figure 1 provides an overview of the irradiated Cu(110) microstructure. Scanning TEM (STEM) high angle annular darkfield (HAADF) micrographs (Figure 1a,b) show the microstructures of the 11 and 110 dpa specimens, respectively, full of voids. The average swelling between the surface and a depth of 1500 nm was calculated to be 1.86 ± 0.36% and 10.6 ± 4.7% at 11 and 110 dpa, respectively. However, it is surprising that voids extend beyond the ion range computed by SRIM at high damage levels, with a notable presence of smaller voids in the injected interstitial region. A similar observation was made by Kim et al. in microstructures containing a large void volume fraction that arose from increasing porosity in the shallower depths [6]. Other studies have observed the presence of defects appearing beyond the SRIM predicted ion range [26,27,28,29,30,31]. Under channeling conditions, ions penetrate deeper into the material due to the reduced stopping power [26,27]. At elevated temperatures, studies concluded that the high mobility of defects in the specimens coupled with the influence of developing stress states in the irradiated region and damage cascade energy dissipation on enhancing defect mobility can give rise to the formation of defects beyond the predicted ion range [28,29,30,31].

**Figure 2:**
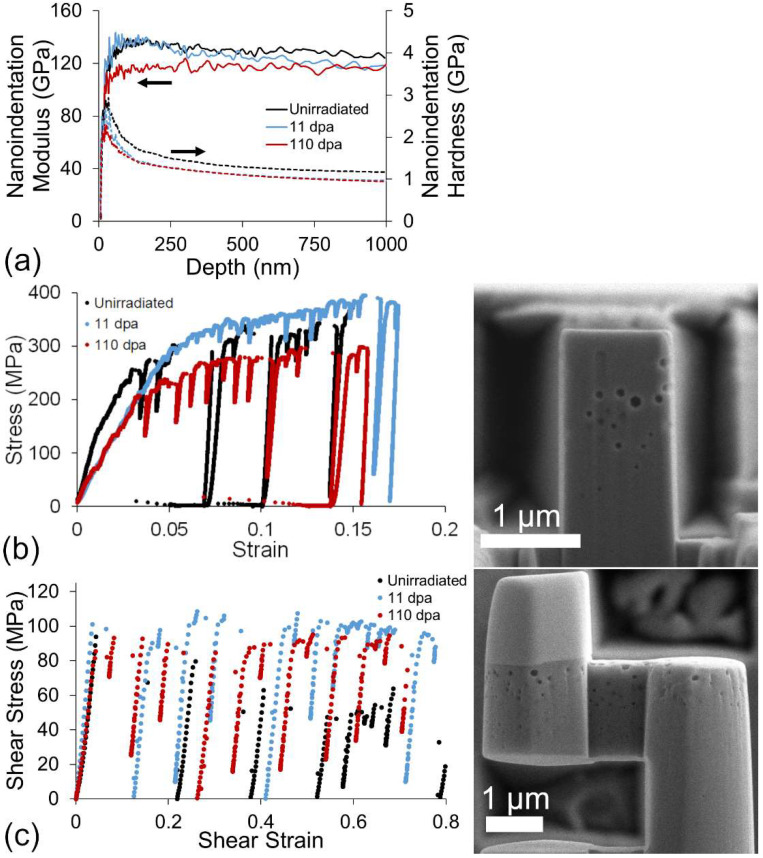
Comparison of the (**a**) nanoindentation, (**b**) micropillar compression, and (**c**) microshear mechanical properties for the unirradiated, 11 dpa, and 110 dpa specimens, respectively. SEM micrographs of a micropillar and microshear test specimen in the 110 dpa sample inset next to the respective mechanical testing results.

Ion-induced void swelling typically induces net positive material flow opposite to the ion incident direction and is correlated to the compressive residual stress induced by void formation [32,33]. Although not directly measured in this study, we can infer from previous studies that the presence of void swelling in the material induces a component of this stress in the irradiated samples. It is important to note, however, that the formation of micropillar and microshear specimens (Figure 2) relieves this stress state. The nanoindentation response, on the other hand, is influenced by any residual stress in the material.

Figure 2 provides an overview of typical material response curves for nanoindentation, micropillar, and microshear compression performed in this study. SEM images of the micropillar and the shear specimen, taken at a tilt of 85°, are provided next to the respective engineering stress-strain plots. Nanoindentation results in a depth region between 50 and 500 nm in Figure 2a show that the measured modulus of the 110 dpa peak specimen is 115 GPa, markedly lower than the unirradiated and 11 dpa peak specimens with measured moduli of 135 GPa. This matches closely with a microstructure that exhibits void swelling of ~20% in this depth region. The trend for nanoindentation hardness, however, decreases after irradiation and does not change significantly after additional irradiation. Microshear and micropillar compressions suggest a different behavior, with a maximum yield strength at 11 dpa, followed by softening at the higher damage level.

**Figure 3:**
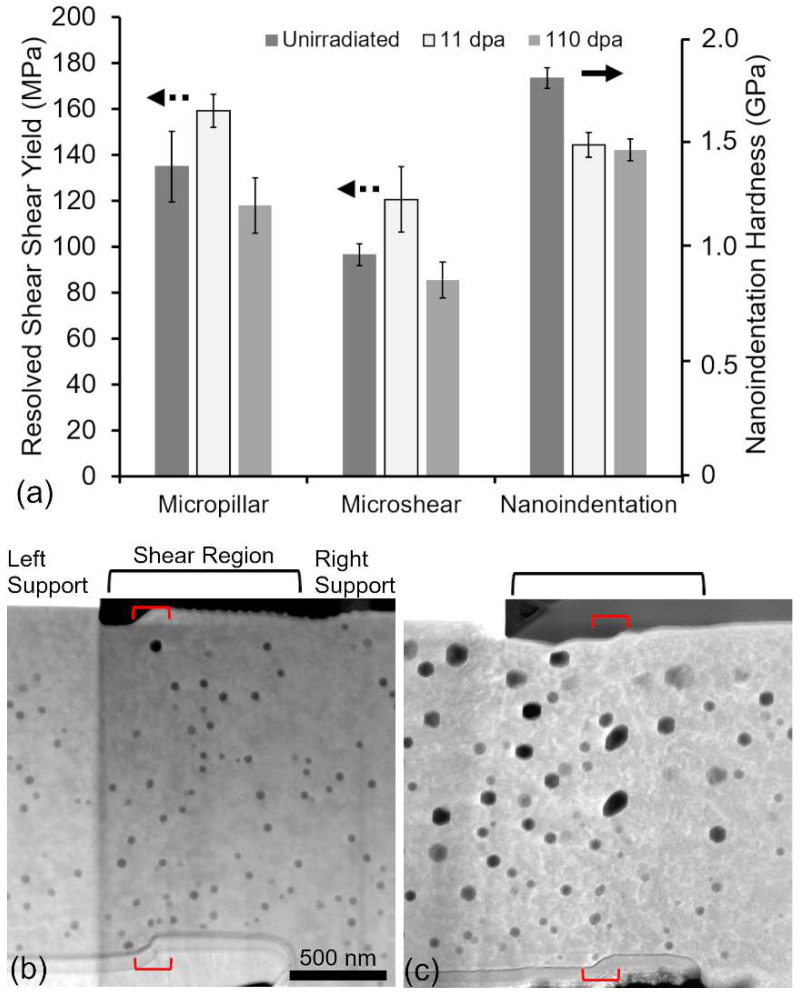
(**a**) Comparison of the resolved shear yield strengths and nanoindentation hardness measured from the unirradiated and irradiated Cu. STEM-HAADF micrographs of (**b**) 11 and (**c**) 110 dpa shear specimens just after yielding.

Figure 3a provides a comparison of the yield strength and nanoindentation hardness obtained from the unirradiated and irradiated Cu samples. Figure 3b,c shows STEM-HAADF micrographs of shear specimens loaded to the first observed strain burst and subsequently unloaded. The red brackets overlaid on the images show that the extent of deformation is confined to a narrow volume in the material. Although difficult to observe in Figure 3b (11 dpa), voids are clearly sheared during deformation and can be easily seen in Figure 3c (110 dpa). Voids in the shear region neighboring this deformation volume do not show visible deformation nor any changes to the shear orientation.

**Figure 4:**
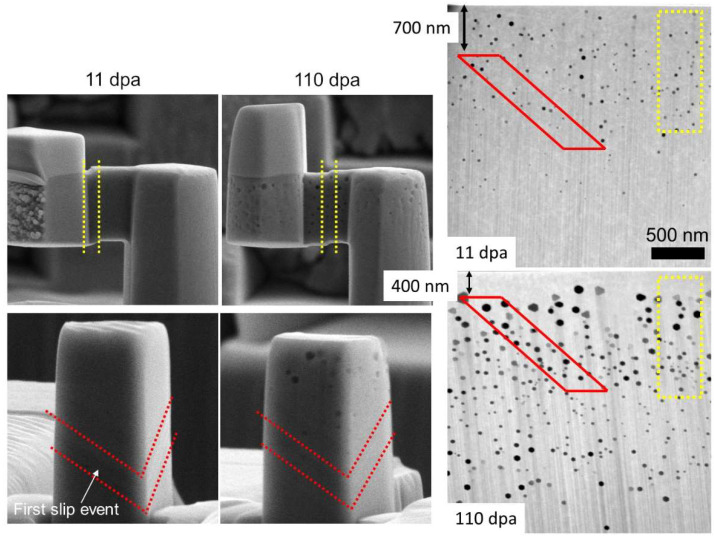
Comparison of the locations where slip traces are first observed in the microshear and micropillar specimens at 11 and 110 dpa.

Microshear specimens fabricated in this study offer a unique advantage over conventional microscale test specimens, such as micropillars or microtensile bars. Here, a single slip system [110](111) can be selectively activated by the microshear structure through a careful selection of shear region orientation. Moreover, the deformation in this system can be confined to a pre-determined portion of the irradiated region to measure an averaged response of irradiation-induced microstructural changes. Such a degree of control is not offered by conventional microscale structures, an example of which is shown in Figure 4.

Figure 3 clearly shows that simple shear testing and micropillar compression result in a similar trend in the resolved yield strength. Nanoindentation shows a notably different trend than either the microshear or micropillar tests. The decrease in hardness observed after irradiation at both damage levels can arise from a number of sources, including annealing, the increase in dislocation content, void swelling, and residual stresses arising from microstructural changes [32,33]. While annealing may account for a part of the decrease in hardness at the high damage level, the discrepancy in behavior is not resolved by this alone, as shown in Supplementary Material Figure S1. At 11 dpa, it is more likely that the presence of irradiation-induced defects helps establish dislocation sources that mediate plastic flow [15,34]. While this has been observed to remove “pop-in” behavior, it may also extend to a reduction in the measured hardness at shallow depths. At 110 dpa, the softening from void swelling, hardening from increasing dislocation content, and the development of a compressive residual stress state all influence the measured hardness. The individual contribution of these to the nanoindentation response is not quantified here, as further experiments to separate the influence of each defect are needed to provide a better understanding.

Despite the similarities in irradiation hardening behavior, it should be noted that the loading configurations for the micropillar and microshear specimens are uniquely different. At low strain values, the micropillar is placed under a uniaxial compression load, while the microshear specimen exhibits a simple shear behavior. The mechanism of deformation for either specimen, however, remains slip via the {111} family of planes. For both specimens, slip initiates at one free surface and terminates at another. The resolved shear stress on the slip plane, however, is different for the different structures. The slip plane for the micropillar compression occurs with a Schmid factor of 0.41 compared to a more direct loading with the microshear specimen for a Schmid factor of ~1. For a more accurate comparison, it is crucial to track the location of the first slip event and associated defect density in that region.

The prominent feature of the microshear specimens is a well-defined slip along a targeted slip plane through the selected portion of the irradiated region at both damage levels. For the micropillar, however, the appearance of the first set of slip traces after yielding varies with damage level. Figure 4 provides a comparison of the slip plane locations on the microshear and micropillar specimens. The red and yellow markers outline the location of the first slip trace for the micropillar and microshear specimens, respectively. Dashed lines are provided on the micromechanical specimens to mark the first slip trace appearance on the specimens observed during the test. Corresponding markers on the cross-section TEM lamella are provided as a guide to understanding ion region and defect differences that the first slip trace encounters between each specimen and damage level. At 11 dpa, the first observed slip traces span a range from 700 nm to 1900 nm below the ion incident surface. At 110 dpa, the region shifts nearer to the surface from 400 nm to 1600 nm. This results in different parts of the irradiated region experiencing yielding at the two damage levels.

Voids act as obstacles to dislocation motion and can serve to harden materials [35]. From a microstructural perspective, an increase in void size and density should result in a monotonic increase in hardness from 11 dpa to 110 dpa. In addition, the dislocation content is also expected to increase monotonically with increasing dose and should compound on any hardening contribution from the presence of voids. This perspective, however, is a bit simplified as the void swelling at 110 dpa is large (greater than 25%) in certain regions. At significant void swelling values, as is the case for the 110 dpa specimen, it is more reasonable to expect softening to occur due to a lack of material (i.e., foam) in the test volume. It is clear from Figure 3 that the softening from large void swelling values dominates the response.

At 11 dpa, the average void size and density in the depth region where slip traces first appear in the micropillar, are 42 ± 2 nm and (4.4 ± 0.61) × 10^14^ cm^−3^, respectively. For the microshear specimen (100 nm to 1500 nm), the void size and density are 40 ± 2.2 nm and (4.3 ± 0.62) × 10^14^ cm^−3^, respectively. If we consider that the hardening introduced by void swelling from the dispersion barrier hardening model is $∆\sigma \sim \sqrt{Nd}$, where *N* is the void density and *d* is the void diameter, then the difference is only ~3%. At 110 dpa, the average void diameter and density for the micropillar slip region are 60 ± 3.6 nm and (3.3 ± 0.36) × 10^14^ cm^−3^, compared to 71 ± 3.4 nm and 2.9 × 10^14^ cm^−3^ ± 0.47 × 10^14^ cm^−3^ for the microshear specimen. At 110 dpa, the difference is even less at 2%. Variations in where slip occurs, while significantly different between the two doses, contribute to a small difference in the observed radiation-induced changes to the mechanical response.

The test specimen geometry must also be considered at the scales investigated. Size effects arising from limited dislocation sources in the test volume are unavoidable at this length scale and have been extensively studied [36,37,38,39]. It has been shown that irradiation can introduce defects that mediate deformation and reduce the influence of size on the strength of micropillar compression [40]. The strained volumes for both the micropillar (2 µm^3^) and microshear (1.5 µm^3^) specimens are similar; therefore, the expectation is that both will be affected similarly, albeit with slight differences in shear yield.

**Figure 5:**
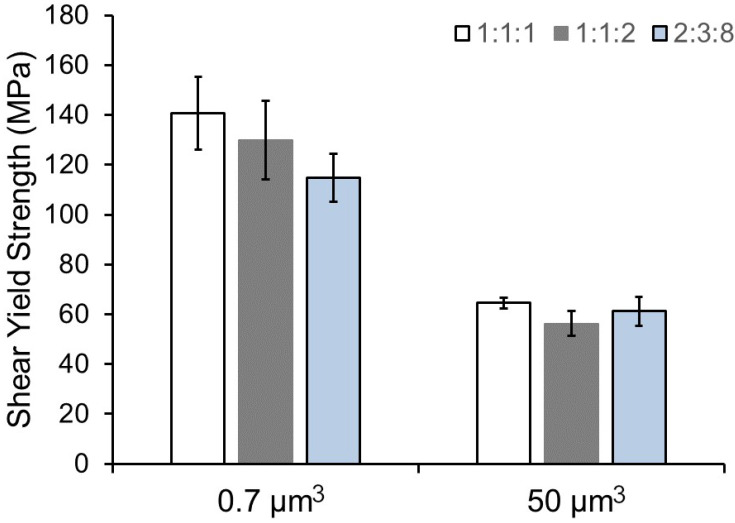
Comparison of the shear yield strength between selected aspect ratios and deformation volumes.

The geometry of the deformed volume may also play a role in any differences observed. Both structures have a deformed region with a 1:1:1.5 (microshear) and 1:1:2 (micropillar) aspect ratio, and no bending (out-of-loading plane deflection) was observed. While micropillar specimens have well-established guidance on aspect ratios [41], microshear specimens have not received similar considerations. Fabrication of shear specimen geometries with varying width:thickness:length ratios shown in Figure 5 reveal a sensitivity to selection. With a fixed deformation volume, larger aspect ratio shear volumes have lower yield stress at the strained volumes used in this study. This may be due to the fact that the effective width of the volume that is deformed during slip in this loading configuration is narrow, as shown in Figure 3b,c. Therefore, the choice of volumes with a very large height to width/thickness ratio is preferred to minimize both bending moments at the supports and size effects at this length scale. In this instance, however, the difference between 1:1:2 and 2:3:8 aspect ratios is ~10%, with the difference diminishing at larger strained volumes.

## 4. Conclusions

The present study showcases an initial attempt to apply microscale shear testing to irradiated materials. The motivation for the current work stems from the need to perform accelerated ion irradiation damage studies and understand the impact of ion irradiation on mechanical performance. A recent study by Eftink et al. demonstrated the use of shear punch testing to probe mechanical property changes on small neutron irradiated disks and provides additional motivation to develop micromechanically accessible shear tests in ion-irradiated materials.

Single crystal Cu specimens were irradiated with 10 MeV Cu ions at elevated temperatures to induce void swelling in the target material. TEM investigations coupled with a machine-learning analytical tool were used to generate statistics over large areas. Microshear, micropillar, and nanoindentation were used to probe the changes in mechanical properties after ion irradiation. Nanoindentation and micropillar compression were selected as they are commonly utilized to probe ion-induced changes to the mechanical properties. Ion-induced void swelling reaches levels of ~2% and ~10% at 11 and 110 peak dpa, respectively. In some regions at 110 dpa, void swelling reached levels in excess of 25%.

Microshear specimens found good agreement with micropillar compression response after irradiation. Both test specimens followed an intuitive expectation from the microstructure. At 11 dpa, hardening was observed, followed by softening at 110 dpa. The discrepancy with nanoindentation results was speculated to arise from a number of sources, including residual stresses, annealing, and an increase in dislocation sources to mediate plastic flow. Further tests are needed to identify the controlling factor for the trend in nanoindentation hardness.

It should be noted that slight differences in the resolved shear stress values—owing to different loading geometries, location of slip initiation, and deformation region geometries—are present between the two test types. These factors, however, do not significantly influence the trend in the measured resolved shear yield. The similarities between micropillar and microshear specimen deformation behavior support the viability of microshear specimens for exploring the radiation response. Further studies are planned to explore the alignment of the microshear response for ion irradiated materials to that observed in shear punch testing with neutron irradiated materials.

## Data Availability

The data presented in this study are available on request from the corresponding author.

## Supplementary Materials

The following supporting information can be downloaded at: https://www.mdpi.com/article/10.3390/ma15124253/s1, Figure S1: Nanoindentation hardness and modulus comparison for as-received and annealed single crystal copper. Sample annealed for same duration as 110 peak dpa irradiation.
