# Supplementary material for: A Novel Microshear Geometry for Exploring the Influence of Void Swelling on the Mechanical Properties Induced by MeV Heavy Ion Irradiation
Source: Materials (Basel). 2022 Jun 15;15(12):4253. doi: 10.3390/ma15124253 (PMC9231319; doi:10.3390/ma15124253)
Supplement: Supplementary file 1 [file materials-15-04253-s001.zip › materials-1728385-supplementary.pdf]

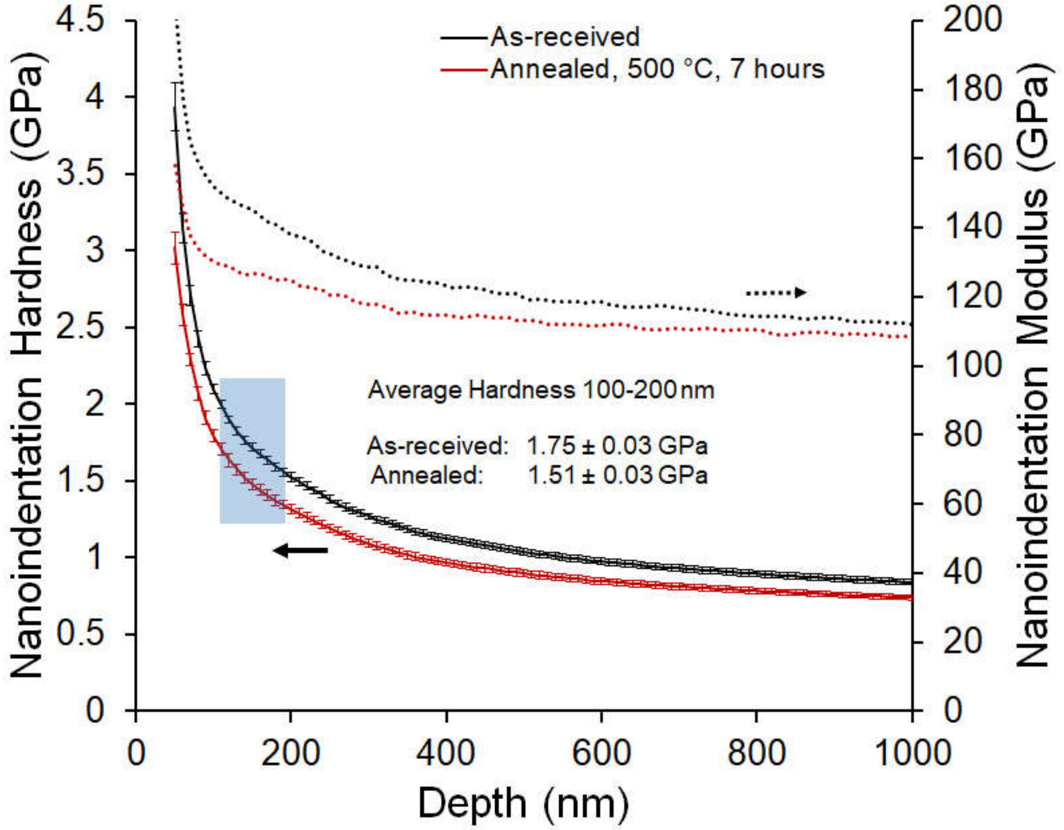

Figure S1 – Nanoindentation hardness and modulus comparison for as-received and annealed single crystal copper. Sample annealed for same duration as 110 peak dpa irradiation.
